# Humoral and cellular immune response in patients with hematological disorders after two doses of BNT162b2 mRNA COVID‐19 vaccine: A single‐center prospective observational study (NCT05074706)

**DOI:** 10.1002/jha2.544

**Published:** 2022-08-30

**Authors:** Elisa Bossi, Andrea Aroldi, Lorenza Maria Borin, Luisa Verga, Diletta Fontana, Federica Cocito, Beatrice Manghisi, Giovanni Rindone, Fabrizio Cavalca, Alessia Ripamonti, Monica Raggi, Sergio Maria Ivano Malandrin, Annalisa Cavallero, Laura Antolini, Diego Bonardi, Rocco Giovanni Piazza, Carlo Gambacorti‐Passerini

**Affiliations:** ^1^ Department of Hematology San Gerardo Hospital Monza Italy; ^2^ Department of Medicine and Surgery University of Milano‐Bicocca Milano Italy; ^3^ Microbiology Laboratory San Gerardo Hospital Monza Italy

**Keywords:** cellular immune response, COVID‐19, hematological disorders, mRNA vaccine, seroconversion

## Abstract

Hematological patients at higher risk of severe COVID‐19 were excluded from the severe acute respiratory syndrome coronavirus 2 (SARS‐CoV‐2) vaccine trials. In this single‐center observational prospective study (NCT05074706), we evaluate immune response in the hematological patients followed at the Hematological Division of San Gerardo Hospital, Monza (Italy) deemed to be severely immunosuppressed after vaccination with two doses of the BNT162b2 vaccine. Anti‐SARS‐CoV‐2 immunoglobulin G titers above the cutoff value of 33.8 BAU/ml were detected in 303 (80.2%) out of the 378 patients enrolled. Patients with lymphoproliferative disorders had a significant lower probability of immunization (43.2% vs. 88.4%, *p* < 0.001). Patients treated with anti‐CD20 showed a significantly lower probability of immunization compared to all other treatments (21.4%, *p* < 0.0001). Among 69 patients who failed seroconversion, 15 patients (22.7%) showed a positive T‐cell response. Patients previously treated with anti‐CD20 were 2.4 times more likely to test positive for T‐cell responses (*p* = 0.014). Within a follow‐up of 9 months from the second COVID‐19 vaccination, symptomatic SARS‐CoV‐2 infections were reported by 20 patients (5.3%) and four of them required hospitalization. Successful serological or T‐cell‐mediated immunization conferred protection from symptomatic COVID‐19. Patients treated with anti‐CD20 who were not seroconverted after vaccination might still be protected from COVID‐19 due to the T‐cell immune response.

## 1 INTRODUCTION

COVID‐19, the disease caused by severe acute respiratory syndrome coronavirus 2 (SARS‐CoV‐2) has been declared pandemic in March 2020. Patients with hematological malignancies have been excluded from the SARS‐CoV‐2 vaccine trials, despite being at higher risk of severe COVID‐19 with a high mortality rate of 30%–37% [1, 2, 3]. However, most health authorities worldwide have designated these patients as a priority for COVID‐19 vaccination even in the absence of efficacy data given their status of highly immunosuppressed patients [4]. Follow‐up studies on seroconversion in cancer patients with COVID‐19 demonstrated that while most will develop antibody response similar to the general population, subgroups of cancer patients with hematologic malignancies, receiving anti‐CD20 antibody therapies and stem‐cell transplantation, exhibit lower rates of seroconversion (70%) in comparison with all other hematologic patients (85%) [5]. However, T‐cell response is induced by natural SARS‐CoV‐2 infection or vaccination and plays a central protective role as it usually does in viral infections. It has been reported that SARS‐CoV‐2‐specific T‐cell responses were inhibited in cancer patients and there is limited information about the T‐cell‐mediated vaccine responses after anti‐CD20 treatment [6].

To gain more insights in the immunogenicity of mRNA vaccines in patients with hematologic malignancies, we analyzed the antibody as well as the T‐cell response after the second dose of the BNT162b2 vaccination in hematological patients with important degree of immunosuppression.

## 2 MATERIALS AND METHODS

### 2.1 Study participants and data collection

All the hematological patients followed at the Hematological Division of San Gerardo Hospital, Monza (Italy), who had been offered vaccination with two doses of the BNT162b2 vaccine (Pfizer‐BioNTech) were recruited to participate in this single‐center prospective observational study (NCT05074706). Only patients deemed to present a moderate to high immunosuppression due to active treatment or type of hematological disorder were enrolled: for example, no patient with chronic myeloid leukemia was included; the selected population represented approximately 15% of all hematologic patients followed at our department. The study was approved by the local Ethical Committee of San Gerardo Hospital of Monza and was conducted according to ethical principles of the Declaration of Helsinki. All patients were provided written informed consent prior enrollment in the study. Hematological disease subtypes were divided as follows: acute leukemia (myeloid or lymphoblastic), myelodysplastic syndrome, myeloproliferative neoplasms (including polycythemia vera, essential thrombocythemia, and myelofibrosis), lymphoproliferative disorder (aggressive or indolent non‐Hodgkin's lymphoma, Hodgkin's lymphoma, and chronic lymphocytic leukemia), multiple myeloma, previous allogenic hematopoietic stem‐cell transplantation (Allo‐HSCT), and “other hematologic disorders” (including immune thrombocytopenic purpura, hemolytic anemia, thrombotic thrombocytopenic purpura, and aplastic anemia). Active therapy was defined as: any patient receiving treatment for underlying hematological disease during or within 1 month before the completion of the vaccination schedule. Treatment regimens included: anti‐CD20 monoclonal antibodies (rituximab, obinutuzumab), Bruton tyrosine kinase inhibitors (BTKI) (ibrutinib), target therapies (ruxolitinib), generic immunosuppression (steroids, azathioprine, cyclosporine, methotrexate, mycophenolate), and other therapies (chemotherapy, immunotherapy, and target therapies different from those specifically categorized). SARS‐CoV‐2 immunoglobulin G (IgG)‐specific antibodies were detected on left‐over biological material (serum or plasma) collected during routine blood tests performed between 30 and 60 days after the administration of the second vaccine dose. Additionally, the functional T‐cell response was analyzed in patients without serological response after two doses of SARS‐CoV‐2 vaccination. Blood samples were processed locally at San Gerardo Hospital.

### 2.2 Serologic testing

SARS‐CoV‐2 IgG was tested on serum or plasma by LIAISON (DiaSorin) SARS‐CoV‐2‐TrimericS IgG assay, a new chemiluminescence immunoassay for the quantitative determination of anti‐trimeric spike protein‐specific IgG antibodies against SARS‐CoV‐2 in human serum or plasma samples. The LIAISON SARS‐CoV‐2 TrimericS IgG assay measures between 4.81 and 2080 binding anticorpal unit (BAU)/ml. Seroconversion is defined as a post‐vaccination SARS‐CoV‐2 IgG antibody titer ≥33.8 BAU/ml. Technical specifications are shown in Table S1.

**TABLE 1:** Patients’ baseline characteristics

Sex, *n* (%)	
Male	200 (49.5)
Female	204 (50.5)
Age (years), *n* (%)	
18–40	20 (5.0)
41–50	45 (11.1)
51–60	79 (19.6)
61–70	148 (36.6)
>70	112 (27.7)
Baseline disease, *n* (%)	
AML/ALL	10 (2.6)
MDS	21 (5.6)
CMPD Ph negative	131 (34.7)
HL/NHL/CLL	81 (21.4)
MM	51 (13.5)
Allo‐HSCT	58 (15.3)
Other^a^	26 (6.9)
Type of stem‐cell transplant, *n* (%)	
Allo‐HSCT	62 (16.4)
ASCT	43 (11.4)
ASCT and Allo‐HSCT	4 (1.1)
Status disease at vaccination, *n* (%)	
Complete remission	206 (54.5)
Partial remission	129 (34.1)
Stable disease	38 (10.1)
Progressive disease	5 (1.3)
Time from last treatment to COVID‐19 vaccine, *n* (%)	
Untreated	26 (6.9)
Active treatment	235 (62.2)
≥6 month–1 year	51 (13.5)
≥1 year	66 (17.5)
Treatment given during vaccination, *n* (%)	
Yes	238 (63.0)
No	140 (37.0)
Absolute lymphocyte count before vaccination, median (range; SD)	1740 (310–211,000; 12,678)
Median time from vaccination to serology (days) (SD)	45.6 (15.2)
Type of therapy during vaccination, *n* (%)	
Anti‐CD20	14 (5.88)
BTKI	19 (7.98)
Ruxolitinib	19 (7.98)
Immunosuppressant drugs	15 (6.3)

Abbreviations: ALL, acute lymphoblastic leukemia; Allo‐HSCT, allogenic hematopoietic stem‐cell transplantation; AML, acute myeloid leukemia; ASCT, autologous hematopoietic stem‐cell transplantation; BTKI, Bruton tyrosine kinase inhibitors; CLL, chronic lymphocytic leukemia; CMPD Ph negative, chronic myeloproliferative disorders Philadelphia negative; HL, Hodgkin lymphoma; MDS, myelodysplastic syndrome; MM, multiple myeloma; NHL, non‐Hodgkin lymphoma; SD, standard deviation.

^a^ Immune thrombocytopenic purpura, hemolytic anemia, thrombotic thrombocytopenic purpura, and aplastic anemia.

### 2.3 Test for T‐cell response

To test cellular immune response, cells from lithium heparinized whole blood were stimulated with SARS‐CoV‐2‐specific peptides, covering domains of the spike and nucleocapsid proteins. SD Biosensor Covi‐FERON Tubes 500 (Table S2) contains Nil (negative control) tube, Mitogen (positive control) tube, two kinds of SARS‐CoV‐2‐specific Spike Protein Antigen tubes (Original SP Antigen and Variant SP Antigen), and Nucleocapsid Protein Antigen tube (NP Antigen tube). Plasma of the stimulated samples were used for the detection of interferon‐gamma (INF‐γ), using an immunofluorescence assay (Table S3). The software provided a test result in 15 min. Samples were considered positive at a concentration of INF‐γ >0.3 IU/ml for each plasma sample.

**TABLE 2:** Probability of immunization in *N* = 378 patients in subgroups defined by type of disease and binomial model on the relative risk of immunization

Disease	Patients (*N*)	Immunized patients (*N*)	Probability of immunization (%)	Relative risk^a^	95% CI of the relative risk^a^		*p*‐Value^b^
AML/ALL	10	9	90	1.12	0.91	1.39	0.286
MDS	21	21	100	Not evaluable			
CMPD Ph negative	131	121	92.4	1.15	1.07	1.24	<0.0001
HL/NHL/CLL	81	35	43.2	0.54	0.42	0.70	**<0.0001**
MM	51	44	86.3	1.08	0.95	1.21	0.231
Allo‐HSCT	58	51	87.9	1.10	0.98	1.22	0.092
Other^c^	26	22	84.6	1.06	0.89	1.25	0.536

Abbreviations: ALL, acute lymphoblastic leukemia; Allo‐HSCT, allogenic hematopoietic stem‐cell transplantation; AML, acute myeloid leukemia; CI, confidence interval; CLL, chronic lymphocytic leukemia; CMPD Ph negative, chronic myeloproliferative disorders Philadelphia negative; HL, Hodgkin lymphoma; MDS, myelodysplastic syndrome; MM, multiple myeloma; NHL, non‐Hodgkin lymphoma.

^a^ Compared to all patients considered in the denominator of the relative risk with absolute risk equal to 80.2% with 95% CI = (75.8%; 94.1%).

^b^On the null hypothesis of the relative risk equal to 1.

^c^Immune thrombocytopenic purpura, hemolytic anemia, thrombotic thrombocytopenic purpura, and medullary aplasia.

**TABLE 3:** Probability of immunization in *N* = 378 patients in subgroups defined by type of treatment and binomial model on the relative risk of immunization

Therapy	Patients (*N*)	Immunized patients (*N*)	Probability of immunization (%)	Relative risk^a^	95% CI of the relative risk^a^		*p*‐Value^b^
None	140	115	82.1	1.02	0.93	1.12	0.603
Anti‐CD20	14	3	21.4	0.27	0.10	0.73	**0.01**
BTKI	19	4	21.1	0.26	0.11	0.63	**0.003**
Ruxolitinib	19	13	68.4	0.85	0.63	1.16	0.316
Immunosuppression	15	11	73.3	0.91	0.67	1.25	0.573
Other^c^	171	157	91.8	1.15	1.07	1.23	<0.0001

Abbreviations: BTKI, Bruton tyrosine kinase inhibitors; CI, confidence interval.

^a^ Compared to all patients considered in the denominator of the relative risk with absolute risk equal to 80.2% with 95% CI = (75.8%; 94.1%).

^b^On the null hypothesis of the relative risk equal to 1.

^c^Chemotherapy, immunotherapy, and target therapies different from those specifically categorized.

### 2.4 Statistical analysis

Descriptive analysis on the characteristics of enrolled patients was obtained by the calculation of mean and standard deviations for continuous variables and by absolute frequencies and percentages for categorical variables. The proportion (rate) of seroconversion observed after 30–60 days from the second vaccine dose was estimated by the 95% two‐sided confidence intervals for one proportion by the exact (Clopper–Pearson) formula. The probability of immunization was related to explanatory variables, and their possible interactions, by multivariable binary risk regression models with risk ratio as effect measure. Comparisons on the distribution of the binary outcome (immunization yes/no) across groups defined by a categorical variable were obtained by chi‐square test. The whole set of patients was considered as benchmark in the denominator of the relative risks. In the subset of patients who did not develop seroconversion, the probability of having a positive cellular immune response was related to explanatory variables (type of disease and treatment) by multivariable binary risk regression models with risk ratio as effect measure. The whole set of patients who did not seroconverted was considered as benchmark in the denominator of the relative risks. Probability of developing subsequent SARS‐CoV‐2 infection was related to possible prior immunization by multivariable binary risk regression models with risk ratio as effect measure, where again the whole set of patients was considered as benchmark in the denominator of the relative risks. The same probability was also related to type of disease and treatment, which in turn could carry the effect of differential probabilities of immunization. Data analysis was conducted by the STATA software 16.0.

The primary end point was antibody response (seroconversion rate) after the second vaccination dose. The secondary end points were subgroup analysis to correlate the rate of seroconversion per type of hematological disease and per specific treatment, cellular immunity among serological nonresponders patients, and efficacy of the vaccination against symptomatic COVID‐19 infection (documented by a positive real‐time polymerase chain reaction [RT‐PCR]).

## 3 RESULTS

### 3.1 Patients characteristics

A total of 404 hematological patients who completed their full vaccination course with BNT162b2 vaccine (Pfizer‐BioNTech) between April and May 2021 were included in the study. The median age of the patient population was 65 years (range 22–86). Male/female ratio was 0.98. Twenty‐six out of 404 of patients (6.4%, 95% confidence interval [CI] 4.4–9.3) tested positive for SARS‐CoV‐2 infection by PCR analysis of nasopharyngeal swaps prior to the first vaccination dose and were excluded from the analysis. Baseline patients’ characteristics are shown in Table 1. Patients deemed to present a moderate to high immunosuppression due to active treatment or type and status of hematological disease itself were permitted to be enrolled. At the time of vaccination, 238 (63%, 95% CI 58.0–67.7) patients were on‐therapy and 140 (37%, 95% CI 32.3–42.0) patients were off treatment. Most patients were in complete or in partial response at the time of enrollment (54.5% and 34.1%, respectively). The timespan between first and second vaccination doses was 21 days for all patients.

### 3.2 B‐cell response following SARS‐CoV‐2 vaccination

Anti‐SARS‐CoV‐2 IgG titers above the cutoff value of 33.8 BAU/ml were detected in 303 patients (80.2%, 95% CI 75.8–94.1) after completion of the two‐dose vaccination course. Median IgG titer was 688 (standard deviation [SD] ±1968.28). Median age of patients with positive anti‐SARS‐CoV‐2 IgG titers was 64 years (range 24–86). Patients with lymphoproliferative disorders presented a significantly lower probability of immunization compared to all other hematological conditions (43.2% vs. 88.4%, *p* < 0.001) (Table 2). Furthermore, among patients with lymphoproliferative disorders, hypogammaglobulinemia did not affect seroconversion (Table S4), whereas a lower antibody response was detected in patients with lymphocytopenia (lymphocyte <1000/mmc) compared to patients with normal lymphocyte count (lymphocyte ≥1000/mmc) (*p* < 0.001) (Table S5).

**TABLE 4:** Probability of Covi‐FERON immunization in *N* = 66 patients in subgroups defined by type of disease and binomial model on the relative risk of immunization

Disease	Patients (*N*)	Immunized patients (*N*)	Probability of immunization (%)	Relative risk^a^	95% CI of the relative risk^a^		*p*‐Value^b^
AML/ALL	1	0	0	Not evaluable			
CMPD Ph negative	7	1	14	0.63	0.10	4.07	0.626
HL/NHL/CLL	40	12	30	1.3	0.69	2.53	0.402
MM	7	0	0	Not evaluable			
Allo‐HSCT	7	2	28.6	1.26	0.36	4.4	0.72
Other^c^	4	0	0	Not evaluable			

Abbreviations: ALL, acute lymphoblastic leukemia; Allo‐HSCT, allogenic hematopoietic stem‐cell transplantation; AML, acute myeloid leukemia; CI, confidence interval; CLL, chronic lymphocytic leukemia; CMPD Ph negative, chronic myeloproliferative disorders Philadelphia negative; HL, Hodgkin lymphoma; MM, multiple myeloma; NHL, non‐Hodgkin lymphoma.

^a^ Compared to all patients considered in the denominator of the relative risk.

^b^ On the null hypothesis of the relative risk equal to 1.

^c^ Immune thrombocytopenic purpura, hemolytic anemia, thrombotic thrombocytopenic purpura, and medullary aplasia.

**TABLE 5:** Probability of immunization in *N* = 66 patients in subgroups defined by type of treatment and binomial model on the relative risk of immunization

Therapy	Patients (*N*)	Immunized patients (*N*)	Probability of immunization (%)	Relative risk^a^	95% CI of the relative risk^a^		*p*‐Value^b^
None	23	4	17.4	0.77	0.28	2.07	0.598
Anti‐CD20	11	6	54.5	2.40	11.9	4.83	**0.014**
BTKI	12	2	16.7	0.73	0.19	2.80	0.65
Ruxolitinib	4	0	0	Not evaluable			
Immunosuppression	4	1	25	1.10	0.19	6.36	0.915
Other^c^	12	2	16.7	0.73	0.19	2.80	0.65

Abbreviations: BTKI, Bruton tyrosine kinase inhibitors; CI, confidence interval.

^a^ Compared to all patients considered in the denominator of the relative risk with an absolute risk equal to 22.7% with 95% CI = (14.6%; 35.5%).

^b^On the null hypothesis of the relative risk equal to 1.

^c^ Chemotherapy, immunotherapy, and target therapies different from those specifically categorized.

The type of treatment regimen at time of vaccination was found to influence the antibody response: patients treated with anti‐CD20 antibodies and BTKI showed a significantly lower probability of immunization in comparison with all other treatments (21.4% vs. 82.6% and 21.1% vs. 84%, respectively, *p* < 0.0001) (Table 3), whereas no difference was observed for patients who received more than three lines of previous therapies compared to patients not heavily pretreated (74.6% vs. 81.3%, *p* = 0.226). The lower risk of seroconversion for patients treated with anti‐CD20 or BTKI was even clearer when analyzing the subgroup of patients affected by lymphoproliferative disorders, in whom a recent treatment with anti‐CD20 or BTKI reduced the likelihood of seroconversion by 74% and 65%, respectively (Table S6).

**TABLE 6:** Probability of developing symptomatic COVID‐19 in *N* = 369 patients in subgroups defined by prior immunization and binomial model of the relative risk of COVID‐19

Immunization	Patients (*N*)	COVID‐19 patients (*N*)	Probability of COVID‐19 symptomatic disease (%)	Relative risk^a^	95% CI of the relative risk^a^		*p*‐Value^b^
None	51	7	13.7	2.53	1.13	5.69	**0.024**
Positive serology	303	12	4.0	0.73	0.36	1.47	0.379
Positive Covi‐FERON test	15	1	6.7	1.23	0.18	8.57	0.834

Abbreviation: CI, confidence interval.

^a^ Compared to all patients considered in the denominator of the relative risk with an absolute risk equal to 5.4% with 95% CI = (3.3%; 8.2%).

^b^On the null hypothesis of the relative risk equal to 1.

### 3.3 T‐cell response following SARS‐CoV‐2 vaccination

Among the 79 patients who failed seroconversion, evaluation of cellular immune response was performed in 69 patients (10 patients did not provide informed consent to Covi‐FERON test) among which three patients (4.4%, 95% CI 1.5–12.0) had prior documented SARS‐CoV‐2 infection and were excluded from the analysis. The mean time lag between second vaccination and T‐cell response assessment was 42.8 days (SD ±15.2). A total of 15 patients (22.7%, 95% CI 14.3–34.2) with no detectable antibodies after the vaccination series showed a positive T‐cell response. Characteristics of *N* = 69 patients tested for T‐cell immune response are shown in Table S7. Patients with lymphoproliferative disorders had a higher percentage of patients with a positive Covi‐FERON test (30%, 95% CI 18.1–45.4), although the difference was not statistically significant (*p* = 0.402) (Table 4). Patients previously treated with anti‐CD20 were 2.4 times more likely to be tested positive for Covi‐FERON than patients treated with other regimens and this difference was statistically significant (*p* = 0.014) (Table 5).

**TABLE 7:** Probability of developing symptomatic COVID‐19 in *N* = 369 patients in subgroups defined by type of disease and binomial model on the relative risk of COVID‐19

Disease/adjustment variable	Patients (*N*)	COVID‐19 patients (*N*)	Probability of COVID‐19 (%)	Relative risk^a^	95% CI of the relative risk^a^		*p*‐Value^b^
AML/ALL	10	0	0	Not evaluable			
MDS	21	0	0	Not evaluable			
CMPD Ph negative	128	6	4.7	0.86	0.36	2.11	0.75
HL/NHL/CLL	75	5	6.7	1.23	0.48	3.17	0.67
MM	51	7	13.7	2.53	1.13	5.69	**0.024**
Allo‐HSCT	58	1	1.7	0.32	0.04	2.33	0.259
Other^c^	26	1	3.8	0.71	0.10	5.08	0.733

Abbreviations: ALL, acute lymphoblastic leukemia; Allo‐HSCT, allogenic hematopoietic stem‐cell transplantation; AML, acute myeloid leukemia; CI, confidence interval; CLL, chronic lymphocytic leukemia; CMPD Ph negative, chronic myeloproliferative disorders Philadelphia negative; HL, Hodgkin lymphoma; MDS, myelodysplastic syndrome; MM, multiple myeloma; NHL, non‐Hodgkin lymphoma.

^a^ Compared to all patients considered in the denominator of the relative risk with an absolute risk equal to 5.4% with 95% CI = (3.3%; 8.2%).

^b^On the null hypothesis of the relative risk equal to 1.

^c^ Immune thrombocytopenic purpura, hemolytic anemia, thrombotic thrombocytopenic purpura, and medullary aplasia.

### 3.4 Efficacy

Within a follow‐up of 9 months from the second COVID‐19 vaccination, symptomatic SARS‐CoV‐2 infections were reported by 20 patients (5.3%, 95% CI 3.5–8.0), among them four patients (20%, 95% CI 8.1–41.6) needed hospitalization. Only two patients treated with anti‐CD20 developed a severe COVID‐19 and both had negative serological status and no T‐cellular response. Patients who tested negative for both humoral and cellular immune response were indeed 2.5 times more likely to develop a symptomatic COVID‐19 (*p* = 0.024) (Table 6). No COVID‐19 deaths were observed among vaccinated patients with hematological malignancies. At latest follow‐up, three patients (0.8%, 95% CI 0.3–2.3) died, and all of them because of progression of their hematological disease. The probability of developing COVID‐19 in the patients according to type of hematological disease and treatment is shown in Table 7. Fully vaccinated patients with multiple myeloma had a higher probability (relative risk [RR] 2.53, *p* = 0.024) to develop a symptomatic COVID‐19 compared to all other hematological disease subgroups (Table 7). However, patients treated with anti‐CD20 or BTKI had no higher risk of developing a severe COVID‐19 compared to all other treatment regimens (*p* = 0.78 and 0.886, respectively).

## 4 DISCUSSION

In our analysis, the possibility of an antibody response to two‐dose vaccination against SARS‐CoV‐2 in hematological patients was 80.2% (95% CI 75.8–94.1), which is higher than the percentage of 65.3% recently reported by a pooled analysis of 22 studies [7]. We aimed to assess those factors which contributed to the impaired antibody response after COVID‐19 vaccination. In terms of disease subgroups, patients with myelodysplastic syndrome, myeloproliferative disorders, and acute leukemia showed the highest response rates (100% [95% CI 83.9–100], 92.4% [95% CI 86.4–96.3], and 90% [95% CI 55.5–99.7], respectively), whereas patients with lymphoproliferative disorders showed the lowest response rate (43.2% [95% CI 33.2–54.7]). These percentages are similar to those previously reported in literature, with better responses seen in myeloid malignancies and lower responses observed in lymphoid malignancies [8]. Nevertheless, these subgroups of patients per type of disease have different sample sizes, so seroconversion rates are hardly comparable. Moreover, in our analysis, the observation of T‐cell response in the absence of seroconversion is 22.7% (95% CI 14.3–34.2), which was similar to approximately 25%, recently reported in literature [7, 9]. An important treatment‐related factor identified in this analysis is active anti‐CD20 therapy. Recent studies on small cohorts of rituximab‐treated patients with immune‐mediated inflammatory disease provided some initial evidence that T‐cell‐mediated immune response is maintained even in the absence of a humoral anti‐SARS‐CoV‐2 response [10, 11]. Even in our study, performed on 378 hematological patients, active treatment with anti‐CD20 monoclonal antibodies exhibited low seroconversion rates (21.4%, 95% CI 4.7–50.8) but relatively high rates of positive Covi‐FERON tests, indicating successful immunization. These serological results are in line with previous findings of reduced response to anti‐SARS‐CoV‐2 vaccines in patients with hematological malignancies after exposure to B‐cell‐depleting agents [9, 12–14]. However, it is interesting that this subgroup of patients without seroconversion, probably due to active anti‐CD20 therapy, showed the presence of a T‐cell immune response in over half of them. Other studies demonstrated the presence of T‐cell response in hematological patients without antibody response [9, 15]. Additionally, in our study, even patients actively treated with BTKI exhibited very low antibody response rates (21.1%, 95% CI 8.5–43.3). This result is in line with previous studies that found an association between blockaded B‐cell receptor signaling and impaired responses to vaccines against influenza and hepatitis B [16, 17, 18]. However, approximately one in five of these patients showed evidence of an active T‐cell response. Another relevant subgroup consists of patients who underwent Allo‐HSCT. We found slightly high seroconversion rate of 87.9% (95% CI 76.7–95), similarly to what is reported in literature [19]. However, the analysis was not able to differentiate the results according to specific type of donor, time elapsed since transplant, conditioning regimen, duration, and type of immunosuppressive therapy. Another important question is the clinical efficacy of the COVID‐19 vaccines in patients with hematological malignancies. Only 5.3% of patients included in this study developed symptomatic infection in the 9 months after the completion of the vaccination series and few of them required hospitalization. According to type of hematological disease, patients with multiple myeloma had a higher probability (RR 2.53, *p* = 0.024) to develop a symptomatic COVID‐19; this result probably depends on the need for almost constant therapy in this subgroup of patients. However, it was not observed in subgroups of patients treated with anti‐CD20 or BTKI, a higher risk of developing symptomatic COVID‐19, compared to all other treatment regimens (*p* = 0.78 and 0.886, respectively) (Table S8). This means that B‐cell‐depleting treatments should not preclude COVID‐19 vaccination, since a robust T‐cell response may have a protective role in the presence of waning or subprotective antibody titers [11].

The greatest limitation of this study is the lack of a large healthy reference control cohort. However, we know that seroconversion rates in non‐immunosuppressed individuals is close to 100% [16]. Furthermore, subgroups of patients with distinct hematological diseases had different sample sizes, so comparison between cohorts may not be very reliable by relative overrepresentation (including myeloproliferative disorders) or underrepresentation (acute leukemia and myelodysplastic syndrome). Rapid recruitment necessary for studies in this evolving pandemic inevitably affected adequacy of stratification of patients. None of the patients was tested for anti‐SARS‐CoV‐2 IgG titers prior to the first vaccination dose, so the number of patients with previous SARS‐CoV‐2 infection could have been underestimated. Furthermore, kinetics of antibody titers was not evaluated, so it might be possible that a proportion of patients with hematological disorders change from seropositive to seronegative status because of a decline in titers over time. Although neutralizing antibody response represents the gold standard for humoral response, we did not measure it because recent studies demonstrated a high degree of correlation between neutralizing antibody titers and IgG antibodies in both convalescent and vaccinated individuals [20]. Moreover, we did not analyze other patient‐specific factors that may have influenced results, such as age, comorbidities, and concomitant treatments. Analysis of immunogenicity of added booster vaccination dose in hematological patients was out of the scope of the present work and will need further evaluation.

## 5 CONCLUSIONS

Overall, these results indicate that 85%–90% of hematological patients considered to be immunosuppressed are able to mount a positive immune response, either serological or cellular based, to SARS‐CoV‐2 vaccination. In addition, patients with recent or ongoing anti‐CD20 treatment who suffer from insufficient humoral immune response after two COVID‐19 vaccinations might still benefit from vaccination due to the cellular immune response, sufficient to ensure a mild course of disease. This observation should be taken into account while planning consistently effective strategy for hematological patients who remain seronegative after two‐dose vaccination. It is possible that patients with hematological malignancies who exhibited a poor humoral immune response may still be protected by a good cellular immune response.

## CONFLICT OF INTEREST

The authors declare they have no conflicts of interest in relation to the work described.

## AUTHOR CONTRIBUTIONS

Elisa Bossi, Andrea Aroldi, Lorenza Maria Borin, Luisa Verga, Laura Antolini, and Carlo Gambacorti‐Passerini designed the study. Monica Raggi, Sergio Maria Ivano Malandrin, Diletta Fontana, and Annalisa Cavallero performed laboratory analyses. Elisa Bossi, Fabrizio Cavalca, Beatrice Manghisi, Giovanni Rindone, Alessia Ripamonti, and Diego Bonardi collected the data. Elisa Bossi, Carlo Gambacorti‐Passerini, Andrea Aroldi, and Laura Antolini analyzed data. Elisa Bossi, Fabrizio Cavalca, Federica Cocito, Rocco Giovanni Piazza, Laura Antolini, Monica Raggi, and Carlo Gambacorti‐Passerini contributed in writing the article. All the authors equally approved the article.

## ETHICS STATEMENT

The protocol of this study was approved by the Institutional Ethics Review Board and the procedures followed were in accordance with the Declaration of Helsinki. This study has been registered in the Clinical Trial Registry (NCT05074706).

## PATIENT CONSENT STATEMENT

Prior to this study, the principal investigator or investigators obtained written informed consent based on patient free will.

## Supporting information



Supporting Information

## Data Availability

All data generated during this study are available upon justified request to the corresponding author. The authors affirm that this manuscript is an honest, accurate, and transparent account of the study being reported and that no important aspects of the study have been omitted.
