# Supplementary material for: Humoral and cellular immune response in patients with hematological disorders after two doses of BNT162b2 mRNA COVID‐19 vaccine: A single‐center prospective observational study (NCT05074706)
Source: EJHaem. 2022 Aug 30;3(4):1201–8. doi: 10.1002/jha2.544 (PMC9538646; doi:10.1002/jha2.544)
Supplement: Supplementary file 1 — Supporting Information [file JHA2-3-1201-s001.docx]

SDC Table 1. Techical specifications of LIAISON® SARS-CoV-2 TrimericS IgG assay.

| Analyte | IgG antibodies to Sars-Cov-2 |
| --- | --- |
| Platform | LIAISON® XL |
| Expression of Results | BAU/ml Quantitative* |
| Test Format | Indirect immunoassay |
| Determinations | 110 tests/integral  50 determinations/control vial |
| Sample Type | Equivalence to serum shown for SST, Lithium heparin and EDTA plasma |
| Sample Storage | 21 days 2-8°C – 48 hours room temperature |
| Sample Volume | 10 µl |
| Time to First Results | 35 minutes |
| Throughput | 171 test/hour XL |
| Clinical Sensitivity (days post PCR) | 98.7% (≥15 days) |
| Clinical Specificity | 99.5% (95% CI: 99.0-99.7) |
| Correlation Microneutralization Test | PPA: 100% (95% CI: 97.8-100.0)  NPA: 96.9% (95% CI: 92.9-98.7) |
| Assay Range | 4.81-2080 BAU/ml |

*WHO International Reference Standard

SDC Table 2. Specifications of SD Biosensor® Covi-FERON Tubes 500.

| Specimen | Whole blood |
| --- | --- |
| Specimen volume | 1 ml |
| Specimen temperature | 2-8°C / 36-46°C |

SDC Table 3. Specifications of SD Biosensor® Covi-FERON FIA (INF-γ).

| Specimen | Plasma |
| --- | --- |
| Test time | 15 minutes |
| Specimen volume | 100 µl |
| Storage temperature | 2-30°C / 36-86°F |

SDC Table 4. Impact of prior hypogammaglobulinemia on seroconversion following SARS-CoV-2 vaccination in patients with lymphoproliferative disorders.

| SARS-CoV-2 serology status, n(%) | With hypogammaglobulinemia | Without hypogammaglobulinemia | P value |
| --- | --- | --- | --- |
| Positive  Negative | 10 (31.3)  22 (68.7) | 22 (51.2)  21 (48.8) | 0.198 |

SDC Table 5. Impact of prior lymphocytopenia on seroconversion following SARS-CoV-2 vaccination in patients with lymphoproliferative disorders.

| SARS-CoV-2 serology status, n(%) | With lymphocytopenia | Without lymphocytopenia | P value |
| --- | --- | --- | --- |
| Positive  Negative | 37 (62.7)  22 (37.3) | 266 (83.4)  53 (16.6) | <0.001 |

SDC Table 6. Binomial model on the relative risk of immunization in subgroups defined by type of disease and treatment.

| Disease | Therapy | Relative risk* | 95% CI of the relative risk* | | p-value§ |
| --- | --- | --- | --- | --- | --- |
| AML/ALL | None, other^Ƨ^ | 1.12 | 0.91 | 1.39 | 0.286 |
| MDS | None, other^Ƨ^ | not evaluable | | | |
| CMPD Ph negative | None, ruxolitinib | 0.83 | 0.61 | 1.13 | 0.239 |
|  | other^Ƨ^ | 1.46 | 1.08 | 1.98 | 0.015 |
| HL/NHL/CLL | None | 0.74 | 0.58 | 0.96 | 0.022 |
|  | anti-CD20 | 0.26 | 0.07 | 0.95 | **0.041** |
|  | BTKI | 0.35 | 0.14 | 0.87 | **0.025** |
|  | ruxolitinib, immunosuppression, other^Ƨ^ | 0.96 | 0.48 | 1.91 | 0.907 |
| MM | None | 1.11 | 0.93 | 1.32 | 0.236 |
|  | other^Ƨ^ | 0.95 | 0.77 | 1.19 | 0.676 |
| Allo-ASHT | None, ruxolitinib | 1.14 | 1.04 | 1.26 | 0.008 |
|  | Immunosuppression | 0.82 | 0.46 | 1.45 | 0.492 |
|  | other^Ƨ^ | 0.73 | 0.41 | 1.29 | 0.275 |
| Other^~^ | None | 1.13 | 0.93 | 1.38 | 0.202 |
|  | Immunosuppression | 0.83 | 0.53 | 1.28 | 0.393 |
|  | other^Ƨ^ | 0.94 | 0.66 | 1.35 | 0.746 |

* compared to all patients considered in the denominator of the relative risk with absolute risk equal to 80.2% with 95%CI=(75.8%; 94.1%); § on the null hypothesis of the relative risk equal to 1; ~ immune thrombocytopenic purpura, hemolytic anemia, thrombotic thrombocytopenic purpura and medullary aplasia; Ƨ chemotherapy, immunotherapy and target therapies different from those specifically categorized. AML=acute myeloid leukemia; ALL=acute lymphoblastic leukemia; MDS=myelodysplastic syndrome; CMPD Ph negative=chronic myeloproliferative disorders Philadelphia negative; HL=Hodgkin lymphoma; NHL=non Hodgkin lymphoma; CLL=chronic lymphocytic leukemia; MM=multiple myeloma; Allo-HSCT=allogenic hematopoietic stem cell transplantation; ASCT=autologous hematopoietic stem cell transplantation.

SDC Table 7. Characteristics of N=69 patients tested for T-cell immune response.

| Sex, n (%)  Male  Female | 36 (52.2)  33 (47.8) |
| --- | --- |
| Age, n (%)  18-40 years  41-50 years  51-60 years  61-70 years  >70 years | 3 (4.4)  4 (5.8)  14 (20.3)  21 (30.4)  27 (39.1) |
| Prior ≥3 terapies, n (%)  Yes  No | 14 (20.3)  55 (79.7) |
| Baseline disease, n (%)  AML/ALL  MDS  CMPD Ph negative  HL/NHL/CLL  MM  Allo-HSCT  other~ | 1 (1.5)  0 (0)  7 (10.1)  42 (60.9)  7 (10.1)  8 (11.6)  4 (5.8) |
| Type of stem cell transplant, n (%)  Allo-HSCT  ASCT  ASCT e Allo-HSCT | 8 (11.6)  6 (8.7)  1 (1.5) |
| Status disease at vaccination, n (%)  Complete remission  Partial remission  Stable disease  Progressive disease | 40 (58.0)  20 (29.0)  6 (8.7)  3 (4.3) |
| Treatment given during vaccination, n (%)  Yes  No | 46 (66.7)  23 (33.3) |
| Time from last treatment to Covid-19 vaccine, n (%)  Untreated  Active treatment  ≥6 month-1year  ≥1 year | 0 (0)  46 (66.7)  19 (27.5)  4 (5.8) |
| Type of therapy during vaccination, n (%)  Anti-CD20  BTKI  Ruxolitinib  Immunosuppressant drugs  Other^Ƨ^ | 11 (23.9)  14 (30.4)  4 (8.7)  5 (10.9)  12 (26.1) |

~ immune thrombocytopenic purpura, hemolytic anemia, thrombotic thrombocytopenic purpura and medullary aplasia; Ƨ chemotherapy, immunotherapy and target therapies different from those specifically categorized. AML=acute myeloid leukemia; ALL=acute lymphoblastic leukemia; MDS=myelodysplastic syndrome; CMPD Ph negative=chronic myeloproliferative disorders Philadelphia negative; HL=Hodgkin lymphoma; NHL=non Hodgkin lymphoma; CLL=chronic lymphocytic leukemia; MM=multiple myeloma; Allo-HSCT=allogenic hematopoietic stem cell transplantation; ASCT=autologous hematopoietic stem cell transplantation; ASCT=autologous hematopoietic stem cell transplantation; BTKI=Bruton tyrosine kinase inhibitors.

SDC Table 8. Probability of developing symptomatic COVID-19 in N=369 patients in subgroups defined by type of treatment and binomial model on the relative risk of COVID-19.

| Therapy/adjustment variable | Patients (N) | COVID-19  patients  (N) | Probability of COVID-19 symptomatic disease (%) | Relative risk* | 95% CI of the relative risk* | | p-value§ |
| --- | --- | --- | --- | --- | --- | --- | --- |
| None | 138 | 3 | 2.2 | 0.40 | 0.12 | 1.33 | 0.135 |
| Anti-CD20 | 14 | 1 | 7.1 | 1.32 | 0.19 | 9.14 | 0.78 |
| BTKI | 16 | 1 | 6.3 | 1.15 | 0.16 | 8.06 | 0.886 |
| Ruxolitinib | 17 | 2 | 11.8 | 2.17 | 0.55 | 8.54 | 0.267 |
| Immunosuppression | 15 | 0 | 0 | not evaluable | | | |
| Other^Ƨ^ | 169 | 13 | 7.7 | 1.42 | 0.72 | 2.78 | 0.309 |

* compared to all patients considered in the denominator of the relative risk with an absolute risk equal to 5.4% with 95%CI=(3.3%; 8.2%); § on the null hypothesis of the relative risk equal to 1; Ƨ chemotherapy, immunotherapy and target therapies different from those specifically categorized. BTKI=Bruton tyrosine kinase inhibitors.

SDC Table 9. Probability of developing COVID-19 in N=369 patients in subgroups defined by type of disease and binomial model on the relative risk of COVID-19.

| Disease | Prior immunization | Patients (N) | COVID-19 (N) | Prob. of COVID-19 (%) | Relative risk* | 95% CI of the relative risk* | | p-value§ |
| --- | --- | --- | --- | --- | --- | --- | --- | --- |
| AML/ALL | NO  YES | 1  9 | 0  0 | 0  0 |  | Not evaluable  Not evaluable | |  |
| MDS | NO  YES | 0  21 | 0  0 | 0  0 |  | Not evaluable  Not evaluable | |  |
| CMPD Ph negative | NO  YES | 6  122 | 1  5 | 16.7  4.1 | 3.08  0.76 | 0.49  0.29 | 19.35  1.97 | 0.23  0.57 |
| HL/NHL/CLL | NO  YES | 28  47 | 4  1 | 14.3  2.1 | 2.64  0.39 | 0.97  0.05 | 7.18  2.86 | 0.06  0.36 |
| MM | NO  YES | 7  44 | 2  5 | 28.6  11.4 | 5.27  2.10 | 1.52  0.83 | 18.33  5.31 | 0.01  0.12 |
| Allo-HSCT | NO  YES | 5  53 | 0  1 | 0  1.9 | Not evaluable | | | |
|  |  |  |  |  | 0.35 | 0.05 | 2.54 | 0.30 |
| Other~ | NO  YES | 4  22 | 0  1 | 0  4.5 | Not evaluable | | | |
|  |  |  |  |  | 0.84 | 0.12 | 5.96 | 0.86 |

* compared to all patients considered in the denominator of the relative risk with an absolute risk equal to 5.4% with 95%CI=(3.3%; 8.2%); § on the null hypothesis of the relative risk equal to 1; ~ immune thrombocytopenic purpura, hemolytic anemia, thrombotic thrombocytopenic purpura and medullary aplasia. AML=acute myeloid leukemia; ALL=acute lymphoblastic leukemia; MDS=myelodysplastic syndrome; CMPD Ph negative=chronic myeloproliferative disorders Philadelphia negative; HL=Hodgkin lymphoma; NHL=non Hodgkin lymphoma; CLL=chronic lymphocytic leukemia; MM=multiple myeloma; Allo-HSCT=allogenic hematopoietic stem cell transplantation.

SDC Table 10. Probability of developing COVID-19 in N=369 patients in subgroups defined by type of treatment and binomial model on the relative risk of COVID-19.

| Therapy | Prior immunization | Patients (N) | COVID-19 (N) | Prob. of COVID-19 (%) | Relative risk* | 95% CI of the relative risk* | | p-value§ |
| --- | --- | --- | --- | --- | --- | --- | --- | --- |
| None | NO  YES | 19  119 | 2  1 | 10.5  0.8 | 1.94  0.16 | 0.49  0.02 | 7.71  1.14 | 0.345  0.067 |
| Anti-CD20 | NO  YES | 5  9 | 1  0 | 20  0 | 3.69 | 0.61 | 22.42 | 0.156 |
|  |  |  |  |  | Not evaluable | | | |
| BTKI | NO  YES | 10  6 | 1  0 | 10  0 | 1.85 | 0.27 | 12.43 | 0.529 |
|  |  |  |  |  | Not evaluable | | | |
| Ruxolitinib | NO  YES | 4  13 | 1  1 | 25  7.7 | 4.61  1.42 | 0.8  0.21 | 26.55  9.78 | 0.087  0.722 |
| Immunosuppression | NO  YES | 3  12 | 0  0 | 0  0 | Not evaluable | | | |
| Other^Ƨ^ | NO  YES | 10  159 | 2  11 | 20  6.9 | 3.69 | 0.99 | 13.69 | 0.051 |
|  |  |  |  |  | 1.28 | 0.63 | 2.6 | 0.502 |

* compared to all patients considered in the denominator of the relative risk with an absolute risk equal to 5.4% with 95%CI=(3.3%; 8.2%); § on the null hypothesis of the relative risk equal to 1; Ƨ chemotherapy, immunotherapy and target therapies different from those specifically categorized. BTKI=Bruton tyrosine kinase inhibitors.

SDC Table 11. Characteristics of N=303 patients with positive anti-SARS-Cov-2 IgG titers.

| Sex, n (%)  Male  Female | 143 (47.2)  160 (52.8) |
| --- | --- |
| Age, n (%)  18-40 years  41-50 years  51-60 years  61-70 years  >70 years | 14 (4.6)  37 (12.2)  62 (20.5)  116 (38.3)  74 (24.4) |
| Prior ≥3 therapies, n (%)  Yes  No | 47 (15.5)  256 (84.5) |
| Baseline disease, n (%)  AML/ALL  MDS  CMPD Ph negative  HL/NHL/CLL  MM  Allo-HSCT  other~ | 9 (3.0)  21 (6.9)  121 (39.9)  35 (11.6)  44 (14.5)  51 (16.8)  22 (7.3) |
| Type of stem cell transplant, n (%)  Allo-HSCT  ASCT  ASCT e Allo-HSCT | 51 (16.8)  38 (12.5)  3 (1.0) |
| Status disease at vaccination, n (%)  Complete remission  Partial remission  Stable disease  Progressive disease | 164 (54.1)  107 (35.3)  30 (9.9)  2 (0.7) |
| Treatment given during vaccination, n (%)  Yes  No | 188 (62)  115 (38) |
| Time from last treatment to COVID-19 vaccine, n (%)  Untreated  Active treatment  ≥6 month-1year  ≥1 year | 23 (7.6)  188 (62.0)  31 (10.2)  61 (20.1) |
| Type of therapy during vaccination, n(%)  Anti-CD20  BTKI  Ruxolitinib  Immunosuppressant drugs | 3 (1.6)  4 (2.1)  13 (6.9)  11 (5.8) |

~ immune thrombocytopenic purpura, hemolytic anemia, thrombotic thrombocytopenic purpura and medullary aplasia. AML=acute myeloid leukemia; ALL=acute lymphoblastic leukemia; MDS=myelodysplastic syndrome; CMPD Ph negative=chronic myeloproliferative disorders Philadelphia negative; HL=Hodgkin lymphoma; NHL=non Hodgkin lymphoma; CLL=chronic lymphocytic leukemia; MM=multiple myeloma; Allo-HSCT=allogenic hematopoietic stem cell transplantation; ASCT=autologous hematopoietic stem cell transplation. BTKI=Bruton tyrosine kinase inhibitors.
